# Insights into Prevention of Health Complications in Small for Gestational Age (SGA) Births in Relation to Maternal Characteristics: A Narrative Review

**DOI:** 10.3390/jcm12020531

**Published:** 2023-01-09

**Authors:** Sebastian Wołejszo, Agnieszka Genowska, Radosław Motkowski, Birute Strukcinskiene, Mark Klukowski, Jerzy Konstantynowicz

**Affiliations:** 1 Department of Pediatrics, Rheumatology, Immunology and Metabolic Bone Diseases, Medical University of Bialystok, University Children′s Hospital, 15-274 Bialystok, Poland; 2 Department of Public Health, Medical University of Bialystok, 15-295 Bialystok, Poland; 3 Faculty of Health Sciences, Klaipeda University, LT-92294 Klaipeda, Lithuania

**Keywords:** small for gestational age (SGA) births, newborn, maternal age, nutrition, lifestyle, education, pregnancy

## Abstract

Small for gestational age (SGA) births are a significant clinical and public health issue. The objective of this review was to summarize maternal biological and socio-demographic factors and preventive strategies used to reduce the risk of SGA births. A literature search encompassing data from the last 15 years was conducted using electronic databases MEDLINE/PubMed, Google Scholar and Scopus to review risk factors and preventive strategies for SGA. Current evidence shows that primiparity, previous stillbirths, maternal age ≤24 and ≥35 years, single motherhood, low socio-economic status, smoking and cannabis use during pregnancy confer a significant risk of SGA births. Studies on alcohol consumption during pregnancy and SGA birth weight are inconclusive. Beneficial and preventive factors include the “Mediterranean diet” and dietary intake of vegetables. Periconceptional folic acid supplementation, maternal 25-hydroxyvitamin D, zinc and iron levels are partly associated with birth weight. No significant associations between COVID-19 vaccinations and birthweight are reported. A midwifery-led model based on early and extensive prenatal care reduces the risk of SGA births in women with low socio-economic status. Major preventive measures relate to the awareness of modifiable and non-modifiable risk factors of SGA, leading to changes in parents’ lifestyles. These data support that education, monitoring during pregnancy, and implementing preventive strategies are as important as biological determinants in risk reduction of SGA births.

## 1. Introduction

Small for gestational age (SGA) births are most commonly defined as a birth weight measure below the 10th percentile for a gender-specific reference population’s gestational age (GA). Furthermore, severe SGA refers to individuals born with a birth weight below the third percentile [1,2]. The causes of SGA can be divided into four categories, of which uteroplacental is regarded the most common. The remaining categories include maternal, fetal, and socio-demographic factors [3]. Within the last few decades, the high prevalence of SGA reported worldwide has been maintained at a similar level. Moreover, the burden of SGA depends on the socio-economic status of a country or region, with the highest prevalence being observed in South Asia, followed by sub-Saharan Africa [4]. An estimated 32.5 million infants were born SGA in low- and middle-income countries. Most of these infants, an estimated 53% (16.8 million), were born in South Asia. Contrastingly, approximately 11% of infants were born SGA in high-income countries [5]. About 20% of newborns are SGA, whereas 25% of deaths are reported among such infants in these parts of the world. This shows that socio-economic inequalities are strongly associated with a higher prevalence of SGA births [3,6,7]. 

The impact of social factors on population health and global health throughout the life course has been widely discussed. This appears to be a significant determinant for reproductive, perinatal, and neonatal health. The relationship between social factors and the problem of SGA births has not yet been largely studied. Therefore, research about SGA in the context of public health could be significant in making decisions concerning interventions for children’s health and well-being, could help to develop preventive strategies, and to reduce morbidity risk in adulthood [3,8,9,10]. The causal pathway between biological variables (the length of gestation, parity, previous stillbirths), demographic characteristics (parental age, marital status, place of residence), social factors (level of education, main source of income) and the risk of SGA has been investigated in various studies [8,11]. Increased awareness of the significance of the abovementioned factors may allow parents to modify lifestyles, and reduce not only the risk of SGA births, but also decrease the prevalence of short-term and long-term complications [3,12,13,14]. However, available data are limited and further exploration of this issue in a more detailed manner is needed.

SGA is also associated with a significantly higher risk of perinatal morbidity and mortality in comparison to newborns representing birth weight appropriate for gestational age (AGA), including either term or preterm neonates [15,16]. Moreover, SGA has been reported to play a role in poor neurocognitive development, perinatal adverse outcomes such as impaired thermoregulation and immune dysfunction, neonatal asphyxia, hypoglycemia, hypocalcemia, polycythemia, hyperviscosity, and certain severe conditions associated with prematurity (necrotizing enterocolitis, respiratory distress syndrome, retinopathy, and bronchopulmonary dysplasia) [3,12]. Taking all the above into consideration, birth weight and the time of delivery are strongly associated with in-hospital costs. Higher costs for hospital-based health care are noted in premature SGA neonates, when compared with those with AGA, in the first year of life after delivery with estimates almost reaching $25,000. Average healthcare-associated costs of full-term SGA neonates are doubled in comparison with neonates with AGA. Increased average costs of hospitalization of pregnant women and delivery of SGA also have been reported. Higher in-hospital costs may be related to increased complication rates, prolonged post-partum hospitalizations, more frequent hospital readmissions, and a need for excessive medical care [17,18]. Thus, knowledge of potential risk factors of SGA births as well as maternal education, appropriate prenatal care, and early access to specialized health care services may alleviate perinatal adverse outcomes resulting from SGA. Therefore, wellness programs providing high-quality education and early access to interdisciplinary health care may improve both the physical and mental health status of pregnant women [19]. There is a core need for health care workers’ extensive education in the realm of SGA in order to identify and reduce potential risk factors of SGA and treat related medical conditions. If the fetus is at increased risk of being born SGA, appropriate preventive measures should be introduced so as to minimize the risk of short-term and long-term complications and perinatal morbidity and mortality. This would additionally optimize costs associated with hospital-based health care [8,15,16,18].

Importantly, SGA neonates are also at an increased risk of several adult comorbidities, that is, an early onset of metabolic disorders, chronic non-communicable diseases such as diabetes type 2, chronic kidney disease, cardiovascular diseases and obesity, especially those born SGA, but with rapid excessive weight gain during early infancy [3,12,13,14,20]. The underlying issue is that infants who gained excessive weight most rapidly are at higher risk of central obesity, impaired glucose tolerance, cardiovascular diseases and metabolic disorders [13,14,21]. According to a hypothesis by Barker, intrauterine growth retardation, low birth weight, and premature birth may potentially lead to the onset of chronic diseases, including hypertension, coronary heart disease, or non-insulin-dependent diabetes in adulthood [22,23].

Recent knowledge suggests a particular need for a closer look at SGA and health-related outcomes and concerns. Because of the importance of the problem, the objective of this review was to summarize maternal biological and socio-demographic factors and preventive strategies used to reduce the risk of SGA births.

## 2. Methods 

The available literature was reviewed using electronic databases such as MEDLINE/PubMed, Google Scholar, and Scopus. Publications from the last 15 years (from February 2008 to October 2022) were analyzed. Our search was limited to relevant articles written in English. Risk factors were stratified according to: biological variables (the duration of gestation, parity, pre-eclampsia, previous stillbirths), social factors (level of education, main source of income) and demographic characteristics (parental age, marital status, place of residence, geographical region). Preventive strategies were divided into the 10 following categories: midwifery care, health promotion programs for smoking cessation, alcohol reduction during pregnancy, folic acid, vitamin D, iron, and zinc supplementation, a well-balanced diet, COVID-19 vaccination and cannabis use during pregnancy. The most significant and high-quality publications focusing on preventive strategies [24,25,26,27,28,29,30,31,32,33,34] are summarized in Table 1.

### 2.1. Biological and Socio-Demographic Characteristics of Parents

Maternal age has already been proven to be related to adverse birth outcomes. Mothers aged less than 24 years old are at increased risk of delivering SGA neonates. Similarly, an opposite tendency is recorded in women aged 35 years and older [35]. Nevertheless, considering both maternal age and parity jointly provides a more comprehensive and justified approach, that is, it seems to be more reliable and accurate in predicting adverse birth outcomes [35,36,37]. Multiparous (parity 2–4), grand multiparous (parity 5–8), and great grand multiparous (parity > 8) women are thought to have a lower risk of all adverse birth outcomes regardless of the age, comparing with primiparous women, whereas a significant decrease in birth weight is observed in primigravidas ≥30 years old and multigravidas ≥35 years old, compared with women aged 25–29 years [35,38]. Perinatal mortality and adverse birth outcomes in primiparous women may be associated with fewer, uteroplacental blood flow and smaller uterine cavity in relation to multigravidas [39].

Pre-eclampsia (PE), a severe multisystem disorder of pregnancy caused by abnormal placental blood flow, develops after the 20th week of pregnancy, and is one of the leading causes of maternal and perinatal morbidity and mortality. There is little evidence of the associations between this serious clinical condition and SGA. It is estimated that PE occurs in 3 to 5% of all pregnancies, and is responsible for approximately 14% of deaths of pregnant women per year, whereas the cause of the disease has not been identified yet [40]. Several hypotheses have been developed regarding the etiology and pathomechanism of PE, including abnormal invasion of the trophoblast into the walls of the spiral arteries in the uterus, resulting in an imbalance between angiogenic and non-angiogenic factors. The deterioration of perfusion and placental blood flow may lead to hypoxemia and suboptimal nutrient supply to the fetal tissues, and may be indirectly associated with intrauterine growth restriction (IUGR) [41]. Therefore, early detection of risk factors and initial symptoms of PE as well as early intervention can minimize the risk of short- and long-term perinatal adverse outcomes, such as IUGR, oligohydramniosis, preterm birth, cerebral palsy, hearing loss, visual impairment, insulin resistance, diabetes mellitus, coronary artery disease or hypertension [40,42]. Moreover, there are some moderate-risk factors of PE, such as nulliparity, maternal age above 35 years, obesity, family history of PE or some socio-demographic factors. Based on the above, some of these factors may coexist, and may possibly confer a cumulative risk of both PE and SGA, even if the evidence is scarce [42]. Thus, maternal characteristics play a crucial role in assessing the risk of both SGA birth and PE.

The occurrence of preeclampsia during pregnancy seems to be one of the major factors significantly increasing the risk of miscarriages and stillbirths. According to recent reports, abovementioned factors contribute to decrease newborn weight [43,44,45]. Furthermore, targeted and increased surveillance should also be provided for women with a past history of miscarriages, abortions, or stillbirths. These incidents should be taken into account when assessing the risk of SGA births, especially in the view of currently available data. Each year, about 73 million abortions take place worldwide [46], and in 2019 an estimated 1.9 million babies were stillborn at 28 weeks of pregnancy or later, with a global stillbirth rate of 13.9 stillbirths per 1000 total births [47]. Thus, based on the compelling evidence, these data may also influence designing health policies and preventive strategies to optimize birth outcomes.

The level of education is one of the most critical social determinants and is widely discussed in the literature [11,48,49]. Low educational attainment is unambiguously a significant risk factor of delivering SGA neonates [11,48]. Furthermore, not only maternal, but also paternal lower educational levels have an impact on an annual household income due to unemployment or limited access to other social resources. Greater household wealth is a modifiable factor improving nutritional status and early access to high-quality care, thereby decreasing the risk of delivering an SGA infant [49]. It has been also demonstrated that education of women may have strong influence on lifestyle and nutritional habits, and thereby, on dietary nutritional intakes during pregnancy (Table 1). Regarding prenatal care, there is a relationship between maternal education and the number of consultations. Mothers with a higher level of education are significantly more likely to have more than 6 prenatal consultations, and the first one took place earlier [48]. The association between the importance of education and maternal as well as children’s health can also be explained as follows. Mothers with a higher level of education tend to be more concentrated on themselves, cope better with daily difficulties related to their families and have more knowledge and better judgment in making decisions about health and prenatal testing. The majority of recent reports suggest that educational level appears to be a strong socio-economic predictor of health status and may affect birth weight [48].

In terms of demographic and environmental factors, several variables are reported and highlighted to be associated with pregnancy, pre- and perinatal complications, fetal status and birthweight. High population density is strongly associated with a higher adjusted odds of a low birthweight. This is why place of residence should be taken into consideration when planning offspring. An upward trend in lower birth weight has been observed in big cities and in sub-regions surrounding large cities [50]. Adverse birth outcomes may also be associated with environmental burdens such as overcrowding, inadequate transport systems, noise pollution, and exposure to the air pollution including heavy metals [50,51,52,53]. Rural areas are associated with higher birth weight despite the fact that rural society commonly has restricted access to high-quality health care, demonstrated lower socio-economic status, and an increased occurrence of maternal smoking, There may be a paradox, on the other hand, as the positive effect may be related to some protective factors such as large accumulation of green areas, less air pollution, and healthier lifestyles [50,54]. Nevertheless, assessing the level of risk for delivering neonates with lower birth weight should include all the above confounders to ensure reliability and trustworthiness. Thus, rural-urban environment variables should definitely not be used alone as predictors for overall adverse birth outcomes.

Moreover, a newly suggested model treats GA at the time of delivery and birth-weight Z-scores as continuous variables incorporating also maternal characteristics and history. This interesting updated methodological approach allows for estimation of risks for SGA birth to be personalized [55]. A decreased birth-weight Z-score is associated with ethnicity, racial origin, smoking, past history of stillbirth, chronic hypertension, and systemic lupus erythematous (SLE), whereas parity (understood as multiparity) seems to be a protective factor [55]. However, the cut-off for GA may be changeable during a pregnancy, whereas the cut-off for birth-weight Z-score might be different according to local resources that may lead to increased risk stratification. Furthermore, in different parts of the world we can observe variations in birth weight related to maternal characteristics, and this factor may possibly limit some generalization of the above models. Consequently, there is a strong need to establish ethnic-specific growth charts so as to avoid an underestimation of the increased risk for birth of SGA neonates [56].

### 2.2. Preventive Strategies

Malnutrition during pregnancy and low pre-pregnancy BMI have significant negative effects on birthweight [57,58,59]. Given that approximately 829 million people worldwide are exposed to hunger, this global problem may also have a deleterious effect on pregnant women in some regions and populations. Moreover, world hunger is currently on the rise and has increased by about 150 million affected people from 2019 to 2022 [60]. Therefore, efforts considering nutritional supplementation programmes should be introduced to minimize the burden of adverse outcomes, including SGA births. Balanced protein supplementation introduced for undernourished pregnant women seems to have a great impact on the birthweight [4,61]. Furthermore, the intake of fruits and fishes plays a significant role as well. According to some reports the intake of fruits above 420 g/day is associated with health benefits, that is, the highest probability of AGA births [62]. In a systematic review, Gete et al. found that a greater supply of vegetables, fruits, whole grains, dairy products, and protein diets may contribute to reducing the risk of SGA. What is more, ‘Mediterranean diets’ mostly comprised of fruits, vegetables, legumes, nuts, cereals, seafood, and milk products are associated with a lower risk of SGA births. A ‘Western diet’ mostly characterized by red and processed meat and fatty dairy products increases the risk of SGA [24]. Taking all the above into consideration, educational programmes should include nutritional guidelines in order to improve the quality of a diet during pregnancy as well as draw attention to adequate weight gain before and during pregnancy. This kind of recommendation, however, may be challenging as dietary patterns and nutritional habits considerably differ across countries and cultures, and consumption of nutrients depends of several factors and, in addition, may be population-specific and variable.

In order to provide homeostasis and beneficial effects on human health, micro- and macronutrient intake should be on an appropriate level while their basic source should come from a well-balanced diet. Daily medical practice shows that dietary supplements are overused, ranging from 78% to 98% across USA, Canada, and Australia [63]. This may be associated with, or reflect, general trends in health behaviors, particularly in Western European countries. Nevertheless, certain supplements taken during pregnancy may decrease the risk of adverse birth outcomes. Based on well-balanced evidence, folic acid takes part in a wide range of chemical reactions and pathways, whereas its deficiency may weaken fetal growth, leading to serious congenital conditions, including central nervous system (CNS) defects. Periconceptional folic acid supplementation has been reported to reassure a significant protective effect not only toward development of a neural tube, but also toward SGA births [64]. In an international prospective cohort study (SCOPE), Bulloch et al. found a positive correlation between a pre-conception folic acid supplementation and SGA births [25]. Moreover, a positive correlation between maternal serum 25-hydroxyvitamin D level, antenatal calcium supplementation, and newborns’ birthweight has been shown [65,66]. The pleiotropic effect of vitamin D during pregnancy has been demonstrated in a number of well-designed studies, although diversified evidence level have been reported. In a systematic review and dose-response meta-analysis of observational studies, Zhao et al. found that maternal 25-hydroxyvitamin D concentration was closely related to SGA births and each 25 nmol/L increase in 25(OH)D was linked with even a 10% risk reduction of SGA [26]. Additionally, SGA neonates have significantly lower levels of serum zinc compared to AGA neonates [67,68]. A systematic review by Wilson et al. observed a correlation between umbilical cord and maternal blood zinc levels with birth weight. The above and other studies show that there is a positive relation between maternal blood zinc concentration and birth weight [27]. However, because of the high heterogeneity of related studies, especially in developing countries where people are at increased risk of zinc deficiency, more research is needed. Despite the fact that iron supplementation during pregnancy may contribute to possible adverse outcomes like gestational diabetes, some studies have shown a positive impact of antenatal iron supply, fetal health and birthweight. Martínez-Galiano et al. found a positive correlation between iron intake during pregnancy and SGA [28]. What is more, in a retrospective cohort study conducted in China, Tao et al. found a linear dose-response relationship between ferritin levels and an increased risk of poor birthweight outcomes [29]. These data suggest that maternal ferritin level may be an additional differentiating factor for risk assessment of SGA.

Furthermore, smoking and alcohol consumption are observed more frequently during pregnancy among not only women with lower education levels, but also among single mothers, strongly indicating that marital status should also be included in risk factors category [69,70]. Thus, financial incentives for antepartum and postpartum smoking cessation should be introduced especially among at-risk groups. Smoking-cessation programmes aimed at pregnant women are said to be highly cost-effective due to the decrease of in-hospital costs associated with prolonged health care and adverse birth outcomes [71,72]. There is a high priority and a rationale to implement the strategy into practice in all societies and populations. Approximately 75% of quitters return to smoking within 6 months after childbirth. Consequently, their children are exposed to passive smoking and are at higher risk of smoking-related illnesses [72]. It is worth emphasizing that electronic nicotine delivery systems increase the occurrence of SGA births [73]. In addition, in a randomized controlled trial carried out by Higgins et al., it was shown that financial incentives added to Best Practice increase smoking cessation among not only antepartum, but also postpartum women, were reducing the risk of SGA and potential adverse outcomes [30,74]. A systematic review and meta-analysis conducted by Baía et al. showed that using cannabis by pregnant women appeared to increase the risk of SGA [31]. Alcohol consumption is also linked with adverse perinatal outcomes. It has been proven that alcohol crosses the placenta and fetal blood alcohol levels reach levels that of maternal blood within 2 h of maternal intake [75]. Fetal alcohol effects have been largely discussed in the literature, and the overwhelming evidence of harmful impact on fetal and neonatal health considerably improved clinical practice and awareness, and also changed social perceptions of the problem. In the view of current knowledge about the adverse effects of alcohol, current recommendations strongly suggest complete abstinence during pregnancy [76]. Thus, importantly, most but not all reports support the commonly held notion, that these associations require further well-designed and ethical research. In retrospective cohort studies Pereira et al. found that an alcohol intake during pregnancy appears to be associated with a lower birth weight. However, in prospective cohort studies and case-control studies the association between maternal exposure to alcohol and birth weight was not observed [32].

What is more, antenatal midwifery care generally consists of strengthening a woman’s ability to care for themselves and educating on how to cope with daily difficulties related to their families [77,78]. A midwifery-led model of care is based on antenatal appointments focused on counselling and education as well as socio-cultural and psychological support [33]. Therefore, midwives, and sometimes district or community nurses, are intended to play an influential role in taking care of pregnant women. Data from British Columbia and the entire Canada collected by McRae et al. show that antenatal care is associated with a lower risk of delivering SGA neonates, especially in women with low socio-economic status [33]. There is also a rationale to encourage women to an early initiation of prenatal care, and to remind them about the importance of follow-up visits during pregnancy. The reasonable explanation suggests that the rates of SGA decline with increasing numbers of prenatal care visits.

The COVID-19 pandemic has contributed to an unprecedented acceleration of advanced research on new generation vaccines [79]. Even though COVID-19 vaccination is fully recommended for pregnant women, among others, most of them remain skeptical. Noticeably, the vast majority of recent scientific papers have not shown a negative impact of either COVID-19 vaccinations or COVID-19 infection during pregnancy on adverse birth outcomes [80,81,82,83]. In this context, Lipkind et al. found that COVID-19 vaccination does not appear to have a positive effect on reducing the risk of SGA births, considering the mRNA COVID-19 vaccine dose or a trimester of getting vaccinated, compared with unvaccinated pregnant women [34]. However, there is a core need to motivate pregnant women to get vaccinated in order to minimize subsequent risks of health complications associated with a COVID-19 infection during pregnancy, such as pre-term birth [82]. Infected pregnant women are at increased risk of an admission to intensive care units, an invasive ventilation extracorporeal membrane oxygenation (ECMO) and death, compared with nonpregnant women with symptomatic infections [34]. It matters particularly for women who are in racial and ethnic minorities, who may be at a higher risk of adverse health complications related to COVID-19 [84]. To sum up, more hard evidence is necessary to determine associations between COVID-19 at pregnancy and neonatal outcomes, particularly birthweight. On the other hand, results of future and ongoing prospective and randomized studies may address the potential role and significance of COVID-19 vaccinations in preventing SGA.

### 2.3. Identification of Pregnancies at Higher Risk of Delivering SGA Newborns

There are many prenatal diagnostic tests and tools that are used to roughly estimate birth weight and identify high-risk pregnancies. The “adequate plus” category of Kotelchuck’s Adequacy of Prenatal Care Utilization (APNCU) index is associated with a higher risk of delivery of SGA newborns that may be useful in differentiating the potential risks of SGA births. The APNCU index is based on the month in which prenatal care was initiated and the number of follow-up visits during pregnancy [8,85]. Moreover, fundal height measurements and routine ultrasound exams during pregnancy are reliable for the screening of fetal growth retardation [5]. The abovementioned examinations are relatively inexpensive and may be widely performed in both high- and low-income countries [86]. To assess the obtained measurements, numerous local charts including international standards have been introduced. 

Among many modern models for an EFW, Hadlock’s equations has been accepted as the most precise and reliable. A cut-off point for EFW below the 10th percentile, including gestation, is typical of SGA fetuses. These outcomes should be compared with standardized fetal growth charts or birth-weight Z-scores referring to raw data of a reference population [87]. Consequently, based on these assessment methods, appropriate medical intervention and management strategies of the SGA fetus may be introduced immediately. However, the diagnostic process is still highly challenging despite the great number of investigations. Moreover, guidelines on diagnosing and monitoring SGA fetuses differ across countries and regions [5,88].

The first-trimester fetal ultrasound scan has been implemented in clinical practice as standard procedure in prenatal care. It is performed routinely between 11^+0^ and 13^+6^ weeks of pregnancy [89]. To establish GA precisely, measurements based on the fetal crown-rump length (CRL) seem to be the most accurate and reliable and should be taken before 14 weeks of pregnancy. The first detailed assessment of fetal size and growth is recommended to be conducted in pregnancy weeks 18–22. The second-trimester antenatal screening includes fetal biometric parameters such as head circumference (HC), abdominal circumference (AC), biparietal diameter (BPD), and femur diaphysis length (FL) in order to produce an estimation of fetal weight (EFW) [3,5]. 

The examinations of blood flow through multiple vessels are widely used to predict the fetal condition, especially in the umbilical artery [90,91,92]. The Doppler velocity measurement is a widely used prenatal examination which was introduced many years ago. Available data suggest that abnormal umbilical artery flow with absent or reversed end-diastolic velocity (AREDV) during pregnancy strongly correlates with a higher risk of increased vascular resistance in the fetoplacental vascular system, which may lead to placental insufficiency [90]. Growth-restricted fetuses with identified AREDV in the umbilical artery are at increased risk for perinatal and long-term complications compared to growth-restricted fetuses with preserved AREDV. Scientific studies performed to date suggest that an umbilical artery Doppler velocimetry evaluation should be routinely performed among women with high-risk pregnancies, such as IUGR, PE or gestational hypertension to minimize the risk of adverse birth outcomes. AREDV in the umbilical artery is associated with intraventricular hemorrhage, bronchopulmonary dysplasia, and perinatal mortality, and appears to be associated with the occurrence of respiratory distress syndrome, necrotizing enterocolitis, and long-term neurodevelopmental impairment [91,92]. Although many reliable and credible studies on the development and function of the fetoplacental circulation have been already conducted, many issues concerning the human fetoplacental vasculature particularly in the context of AREDV have not been fully understood.

## 3. Summary and Conclusions

Major preventive measures relate to awareness of modifiable and non-modifiable risk factors of SGA, leading to changes of parents’ lifestyles. A ‘Mediterranean diet’ and well-balanced diet including vegetables, fruits, whole grains, dairy products and proteins have a positive impact on birth weight and should be included in educational programmes alongside with adequate maternal weight gain before and during pregnancy. Periconceptional folic acid supplementation has a significant protective effect toward SGA births. Maternal serum 25-hydroxyvitamin D, zinc, and iron levels are associated with birth weight, although available evidence is inconsistent. The maternal ferritin level may be considered as an additional differentiating factor for risk assessment of SGA. Single mothers are regarded to have a higher tendency to smoking and alcohol consumption, as such, financial incentives for antepartum and postpartum smoking cessation should be introduced as a generally recommended preventive measure. A midwifery-led pregnancy model is associated with a lower risk of delivering SGA neonates, especially in women with low socio-economic status. Physicians and health care professionals should encourage women to seek early initiation of prenatal care, and remind their patients of the importance of follow-up visits during pregnancy. SGA rates essentially decline with increasing numbers of prenatal care visits. There is a need for further research on the effect of COVID-19 vaccination on adverse birth outcomes due to the variety of COVID vaccines available on the market, and limited evidence. All these observations may significantly help in making optimal clinical decisions.

## Figures and Tables

**Table 1:** An overview of the available literature on preventive strategies related to small for gestational age (SGA) births.

First Author and Year of Publication	Study Design	Number of Subjects	Study Period	Significance
Gete, 2020 [24]	Systematic review	Included articles (40): Dietary patterns (22), Foods (13), Food and nutrients (5)	Publications from February 2002 to August 2018	High intake of vegetables, fruits, whole grains, dairy products, and protein may reduce the risk of SGA births. “Mediterranean diets” were associated with a lower risk of having an SGA infant. A “Western diet” (high-fat dairy products, red and processed meat) may increase the risk of SGA births.
Bulloch, 2020 [25]	International prospective cohort study	A total of 5606 women were recruited as part of the Screening for Pregnancy Endpoints (SCOPE) international prospective multi-center cohort study: New Zealand, Australia, United Kingdom and Ireland.	Participants were recruited between 2004 and 2011 from: Auckland (NZ), Adelaide (Australia), Cork (Ireland), London, Leeds and Manchester (UK)	Pre-conception folic acid supplementation was associated with a lower risk of SGA (aOR = 0.82, 95% CI 0.67 to 01.00, *p* = 0.047)
Zhao, 2022 [26]	Systematic review and dose-response meta-analysis of observational studies	The relation between maternal 25(OH)D concentrations and risk of SGA was evaluated in 37 studies, comprising 53,000 participants and 5098 cases. Out of 37 articles, 21 studies were included in the dose–response analysis for SGA.	Years of publication spanning 2010–2020	Maternal 25-hydroxyvitamin D concentration is closely related to SGA births (RR = 0.61; 95% CI 0.49 to 0.76). Each 25 nmol/L increase in 25(OH)D was linked even with a 10% risk reduction of SGA (RR = 0.90; 95% CI 0.84 to 0.97).
Wilson, 2016 [27]	Systematic review	A total of 67 studies met the inclusion criteria, including 29 on SGA/LBW (human prospective cohorts, case-control, longitudinal and cross-sectional studies).	Year of publications related to SGA spanning 1978–2015.	A potential association between maternal dietary zinc intake and infant birthweight
Martínez-Galiano, 2019 [28]	Case-control study	A total of 533 cases were selected: 79 from University of Jaen Hospital, 369 from University of Granada Hospitals, 46 from Ubeda Hospital, 39 from Poniente Hospital.	Case and control groups were enrolled from 15 May 2012, through 15 July 2015 in Spain.	Women receiving iron supplementation >40 mg/day appears to be protective versus women not taking supplements (aOR = 0.64, 95% CI 0.42 to 0.99).
Tao, 2022 [29]	Retrospective cohort study	Total of 3566 pregnant women were included in the study.	Medical information of pregnant women collected from January 2014 to September 2021 at the Zhongnan Hospital of Wuhan University, China.	A linear relationship between maternal ferritin levels and an increased risk of SGA was found (*p*-trend = 0.04), with adjusted OR = 1.87 (95% CI 1.38 to 2.54) for SGA with an increase in Ln-ferritin concentrations per unit.
Higgins, 2022 [30]	Randomized controlled trial	A total of 169 women assigned to best practices (BP) or BP plus financial incentives (BP + FI) for smoking cessation available through to 12 weeks postpartum. A third condition included 80 never-smokers sociodemographically-matched to women who smoked.	Study in Burlington, Vermont, USA, January 2014 through January 2020.	Financial incentives added to Best Practice increase smoking cessation among not only antepartum, but also postpartum women, reducing the risk of SGA and potential adverse outcomes connected.
Baía, 2022 [31]	Systematic Review and Meta-analysis	A total of 32 studies were included: 26 cohort studies: (retrospective [17], prospective [8], prospective multicenter [1]), 4 cross-sectional studies, 1 case-control study, 1 could not be classified. 1 of presented data evaluated SGA.	Restricted to studies published after 2000: United States (22), Australia (4), Canada (3), Czech Republic (1), France (1).	Pregnant women using cannabis are at increased risk for SGA (aOR = 1.47; 95% CI 1.38 to 1.58).
Pereira, 2019 [32]	Systematic Review and Meta-Analysis	A total of 39 studies were included: 15 retrospective studies, 20 prospective cohort studies, and 4 case-control studies.	Restricted to studies published between 1980′s and 2016: Americas (21), Europe (12), Asia (3), Oceania (2), Africa (1).	Retrospectives cohort studies found that alcohol intake during pregnancy appears to be associated with a lower birth weight. However, in prospective cohort studies and case-control studies the association between maternal exposure to alcohol and birth weight was not observed.
McRae, 2018 [33]	Retrospective cohort study	A total of 57,872 pregnant women were included: Who had a low socio-economic status; Who carried a singleton fetus; Who had low to moderate medical/obstetric risk; and Who received medical insurance premium assistance.	Pregnant women who delivered between 2005 and 2012 from British Columbia and Canada.	Antenatal midwifery care is associated with lower risk of delivering SGA neonates versus general practitioner (aOR = 0.71, 95% CI 0.62 to 0.82) or versus obstetrician care (aOR = 0.59, 95% CI 0.50 to 0.69), especially in a group of women with a low socio-economic status.
Lipkind, 2022 [34]	Retrospective cohort study	Single-gestation pregnancies (46,079) with estimated start or last menstrual period between May and October 2020 were included.	A total of 10,064 pregnant women (21.8%) received ≥1 COVID-19 vaccine doses during pregnancy between December 2020 and July 2022.	COVID-19 vaccination in pregnancy was not associated with SGA at birth (aHR = 0.95; 95% CI 0.87 to 1.03).

Abbreviations: aOR—adjusted odds ratio; aHR—adjusted hazard ratio; BP—best practice; CI—confidence interval; FI—financial incentives; LBW—low birth weight; OR– odds ratio; RR—relative risk; SCOPE—Screening for Pregnancy Endpoints.

## Data Availability

No new data were created or analysed in this study. Data sharing is not applicable to this article.
